# Gene-metabolite interactions in aortic dissection: Insights from metabolic and single-cell analyses

**DOI:** 10.1097/MD.0000000000045846

**Published:** 2025-11-21

**Authors:** Chen Yao, Geng Wang, Quanhui Wu, Yifeng Pan, Zhiqiang Chen, Jinming Guo, Chunming Lin, Yitong Peng, Xiaohu Li

**Affiliations:** a Department of Vascular Surgery, The First Affiliated Hospital of Sun Yat-sen University, Guangzhou, Guangdong, China; b Vascular Intervention Department, Zhongshan Hospital of Traditional Chinese Medicine Affiliated to Guangzhou University of Traditional Chinese Medicine, Zhongshan, Guangdong, China; c Science and Education Section, The Eighth Clinical Medical College of Guangzhou University of Chinese Medicine, Foshan Hospital of Traditional Chinese Medicine, Foshan, Guangdong, China.

**Keywords:** aortic dissections, cholesterol metabolism, metabolism, molecular mechanisms, smooth muscle cells

## Abstract

Aortic dissection (AD) involves complex interactions among amino acid, glucose, and lipid metabolism, exacerbating aortic inflammation and extracellular matrix (ECM) degradation, coupled with smooth muscle cell (SMC) dysfunction (phenotypic alteration, aging, apoptosis). To explore AD pathogenesis, we integrated single-cell RNA sequencing (scRNA-seq), metabolomics, machine learning, and Mendelian randomization to investigate SMC changes and gene-metabolite interactions. ScRNA-seq data (GSE213740, GSE155468) were analyzed for cell clustering and pseudo-time trajectories via Seurat and Monocle2. Metabolomics (9 samples: 6 AD, 3 controls) and machine learning validated key genes/metabolites, with Mendelian randomization assessing causal links. Nine cell subsets and 2000 variable genes were identified, with SMCs central to AD via cholesterol metabolism. APOE and PLTP were key genes; metabolomics highlighted cholesterol esters (CEs) and triglycerides (TGs) as critical metabolites. Machine learning confirmed APOE/PLTP’s high predictive accuracy (AUC: 0.796–0.989). Mendelian randomization linked elevated CEs and TGs to increased AD risk (IVW: *P* = .04 and *P* = .02, respectively). This study establishes a gene-metabolite network where APOE and PLTP regulate CEs/TGs, influencing SMC function and AD progression, offering potential therapeutic targets.

## 1. Introduction

Aneurysms of the aorta are characterized by permanent dilation and weakening of the aortic vascular segment, and they are categorized into thoracic aortic dissections (ADs) and abdominal ADs.^[1]^ Although they are relatively rare, with an estimated prevalence of only 2.79 cases per 100,000 people,^[2]^ aneurysms can lead to life-threatening complications such as acute ADs and rupture.^[3]^

Research has confirmed that amino acid metabolism, glucose metabolism, and lipid metabolism play critical roles in the pathogenesis of ADs.^[4]^ Amino acid metabolism affects aortic inflammation, with tryptophan and taurine being examples of metabolites that can modulate the activation and aggregation of inflammatory cells. Inflammation is a significant mechanism in the development of ADs, as it can lead to the release of various inflammatory mediators and proteases, such as matrix metalloproteinase 8 (MMP-8), which promotes the destruction of the aortic wall and inflammation.^[5]^ Macrophages can also affect the remodeling and destruction of the extracellular matrix (ECM), thereby influencing the progression of ADs by controlling ECM remodeling and modulating the inflammatory response.^[6]^

Disorders in glucose and lipid metabolism can lead to the production of a large amount of oxidative stress and pro-inflammatory factors, such as interleukin-6 (IL-6) and monocyte chemoattractant protein-1 (MCP-1), which in turn induce the destruction of the ECM. ECM destruction is also a significant mechanism in the pathogenesis of ADs. The ECM, composed of collagen, elastin, and fibronectin, among other components, constitutes the structural support of the vascular wall.^[7]^ In the case of ADs, the destruction of the ECM is likely influenced by various inflammatory cells and members of the matrix metalloproteinase (MMPs) family, which can degrade the ECM structural proteins and thus disrupt the integrity of the vascular wall.^[8]^ The destruction of the ECM is also affected by the activity of a series of proteases and protease inhibitors. Elevated activity of proteases such as MMPs leads to ECM degradation, which can result in weakened and expanded arterial walls. On the other hand, tissue inhibitors of metalloproteinases (TIMPs) can inhibit the activity of MMPs and other proteases, maintaining the stability of the ECM.^[9]^

SMC dysfunction is a key mechanism in the development of ADs. In the early stages of AD progression, SMCs may undergo phenotypic transformation, losing their original function and morphology, and secreting factors that promote cell proliferation and degradation of collagen, leading to structural destruction and increased vascular wall fragility.^[10]^ The aging of SMCs can lead to apoptosis, which in turn stimulates calcification and exacerbates inflammation, causing circular mechanical damage to the vascular wall.^[11]^ The expression of Nox4 in SMCs can affect the development of ADs by regulating the expression of osteopontin and other proteins. The immune system, through regulating inflammatory responses and cytokine production, can also affect the expression and function of Nox4 in SMCs.^[12]^ Oxidative stress can activate enzymes such as NADPH oxidase and iNOS in SMCs, promoting the production of superoxide radicals and other reactive oxygen species, causing oxidative damage to enzymes and DNA within the SMCs, triggering inflammatory responses and apoptosis.^[13]^ This can lead to phenotypic transformation, loss of normal contraction function, and a shift towards cell proliferation and migration. This phenotypic transformation contributes to ECM destruction, promotes remodeling and expansion of the arterial vascular wall, and ultimately results in the formation of ADs.^[14]^

Clinical and preclinical evidence confirms inflammation as a non - negligible contributor to AD progression: activated macrophages, a central inflammatory cell type, amplify aortic wall destruction by secreting pro - inflammatory cytokines and matrix metalloproteinases (MMPs), with studies showing a direct correlation between macrophage infiltration and AD severity.^[15]^ Clinically, a novel inflammation - based risk score – incorporating markers like C - reactive protein and interleukin-6 – has been validated to predict postoperative mortality in acute type A AD, further underscoring inflammation’s prognostic and pathogenic relevance.^[16]^ This inflammatory cascade is tightly intertwined with metabolic and genetic pathways: dysregulated amino acid, glucose, and lipid metabolism not only fuels oxidative stress and pro - inflammatory factor production (e.g., IL-6, MCP-1) but also modulates the activity of inflammatory cells and ECM - degrading enzymes.^[4,8]^

Notably, the genes and metabolites we focus on – APOE, PLTP, cholesterol esters, and triglycerides – are inherently linked to inflammatory processes. APOE exhibits isoform - specific regulation of inflammation: in vitro studies show APOE ε2 and ε3 isoforms suppress pro - inflammatory cytokine release in glial and neuronal cells, while ε4 exacerbates inflammatory responses and cellular toxicity^[17]^; extrapolating to vascular biology, this suggests APOE may tune AD-related aortic inflammation by modulating macrophage activation or VSMC inflammatory phenotypes. PLTP, meanwhile, is well-established in atherosclerosis – a disease sharing AD’s chronic inflammatory hallmark – where it regulates lipid transport and macrophage foam cell formation, indirectly promoting inflammatory mediator release and vascular wall injury.^[18]^

To further explore the impact of changes within SMCs on AD development, we conducted single-cell data analysis and combined it with metabolomics data to delve deeper into the specific pathways and mechanisms that influence the occurrence and progression of ADs.

## 2. Methodology and materials

### 2.1. Single-cell data analysis

The scRNA-seq dataset GSE213740 for AD, including single-cell sequencing expression profiles of 3 healthy controls and 6 AD patients, was processed by Zhang et al, and the GSE155468 dataset was downloaded from GEO database (https://www.ncbi.nlm.nih.gov/geo/). The main processing of scRNA-seq data was performed using the R software Seurat (version 4.0), including data quality control (QC), evaluation of cell quality and gene expression quality. Subsequently, the NormalizeData function in Seurat was used to normalize gene expression, the ScaleData function was used for normalization of gene expression, and the FindVariableFeatures function was used to identify highly variable genes. Then, the RunPCA function was used for dimensionality reduction to achieve cell clustering and visualization.^[19]^

### 2.2. Biological function enrichment

In this study, we used the clusterProfiler package to conduct GO (Gene Ontology) analysis on the identified differentially expressed genes, performed functional annotations and classified them into different functional categories such as biological processes (BPs), molecular functions (MFs), and cell components, while identifying enriched categories to understand the BPs these genes participate in.^[20]^

Additionally, we utilized the clusterProfiler package to analyze differentially expressed genes and metabolites using KEGG (Kyoto Encyclopedia of Genes and Genomes) analysis, examining their enrichment in pathways in the KEGG database, identifying genes and metabolites enriched in specific biological pathways, and investigating their roles in BPs.^[21]^

In order not to miss metabolic substances with differential expression that are not significant but have important biological significance, we also performed metabolic set enrichment analysis on the differential metabolites, with a setting of 84 human metabolic pathways in the KEGG database and specific biological fluids and blood metabolites related to diseases with biological significance (339 in total). Each metabolic set represents a certain biological function, the metabolomics data is enriched into these metabolic sets, and significant metabolic sets are found.^[22]^

### 2.3. Pseudo-time series analysis

Pseudo-time series analysis was conducted on single-cell data using the Monocle2 package. The steps included data preprocessing, filtering, normalization, and dimensionality reduction. Then, using Monocle2 to automatically identify the main changes in the single-cell data, sorting cells according to these patterns, and constructing a timeline of cells in the pseudo-time, finally getting each cell’s position in the pseudo-time, further inferring the cell’s development state and transcriptional regulatory network. At the same time, through the analysis of the gene expression patterns and functional annotation on the timeline, important transcriptional regulatory pathways, BPs, and key regulatory factors can be identified.

### 2.4. Metabolomics analysis

In order to further clarify the role of metabolites in aneurysms, we performed untargeted metabolomics analysis on 9 patients, including 6 in the AD group and 3 in the CON group.

Multivariate statistics were used to analyze the metabolic groups of the 2 groups of patients. Firstly, the principal component analysis (PCA) of the high-dimensional complex data was carried out, and through orthogonal transformation, the variables that may be correlated were converted into a set of linearly uncorrelated variables, and the original data was compressed into n principal components to describe the characteristics of the original data set. PCA was performed using the built-in statistical function prcomp in R software (www.r-project.org/).

To prevent the occurrence of false negatives in groups with relatively low correlation, orthogonal partial least squares discriminant analysis (OPLS-DA) was performed on the metabolic groups of the 2 groups of patients. Firstly, a PLS model was established to describe the linear relationship between the independent variables and dependent variables. Then, the metamodel was found by minimizing the covariance between the independent variables and dependent variables, and the principal components that explain the maximum difference between the independent variables and dependent variables were found by iteration. Then, the contribution rate of the independent variables (also known as VIP value) was calculated, and the variables with VIP value >1 were selected as features for subsequent classification. Subsequently, the X matrix information was decomposed into 2 classes related to Y and unrelated to Y, the differences were removed, and the differential variables were screened. OPLS-DA was carried out on the data after log2 transformation of the original data and centralized processing.

Based on the OPLS-DA results, the multivariate analysis OPLS-DA model’s variable importance projection (VIP) was obtained as the standard, and the metabolites with differential expression between different species or tissues were screened out. If the biological repetition is < 3, the differential screening is based on the fold change value.

At the same time, the Pearson analysis was used to calculate the correlation between metabolites. First, the covariance of variables was calculated, divided by the strength and direction of the linear relationship between variables. Then, to determine the confidence of the correlation coefficient, the correlation test was carried out to determine whether the correlation coefficient is significantly different from 0, thus confirming whether there is a significant linear correlation between variables. Finally, the top 50 metabolites with significant correlation were selected for visualization based on the *P*-value.

### 2.5. Data acquisition and machine learning validation

The dataset GSE190635 provided by Wang, Fan et al and the dataset GSE202047 were downloaded from GEO database (https://www.ncbi.nlm.nih.gov/geo/). The array was used GPL570 [HG-U133_Plus_2] Affymetrix Human Genome U133 Plus 2.0 Array and GPL13534 Illumina HumanMethylation450 BeadChip. The GSE190635 dataset includes 4 AD patients and 4 healthy individuals, and the GSE202047 dataset includes 8 AD patients. After the probe sequences of the 2 datasets were filtered, invalid probes were removed, and the mean was chosen as the gene expression level after the application of multiple probes to the same gene, the expression profiles were generated. Subsequently, the expression profiles of the 2 key genes APOE and PLTP were read out, forming a prediction model for AD. Then, the model was tested using 4 machine learning algorithms, Logistic Regression, multilayer perceptron (MLP), support vector machine (SVM), and k-nearest neighbor algorithm (K-Nearest Neighbors, KNN).

### 2.6. Mendelian randomization analysis

We conducted Mendelian randomization (MR) analysis for the relationship between differential metabolites and aneurysms. In order to avoid linkage disequilibrium, we took the standard of kb = 10,000 and r2 = 0.001 when aggregating SNPs. At the same time, *P* < 5 × 10-5 was set as the threshold for genome-wide significant. And palindrome SNPs were removed.

The MR analysis mainly employed the inverse variance weighted model (IVW). IVW utilizes the effect size and standard error (or inverse variance) of each genetic variant site to weigh the effect size of each position. By allocating relative weights, IVW reduces estimation bias caused by heterogeneity and provides a comprehensive estimation of the effect size at multiple sites.^[23]^

We also applied 4 statistical methods: the weighted median estimator model (WME), weighted model-based method (WM), MR-Egger regression model (MER), Simple mode (SE). WME can calculate the median effect size of the site, weigh it according to the standard error, and deal with outliers. WM calculates the median effect size of multiple genetic variant sites, and combines them using weighting to obtain the estimate value of the comprehensive effect. MER combines the concept of Egger regression, used to evaluate the bias and symmetry of causal effect estimation. SE can extract basic genetic information and understand the basic genetic features such as the frequency distribution of genotypes and the correlation between genotypes and phenotypes directly. In addition, we also used the Harmonize to remove SNP with incompatible alleles and palindromic SNP, mainly using IVW analysis method, adopt heterogeneity test and MR-Egger regression test. The test results for cholesterol ester and aneurysms were *P*-values of .58 and .80, and for triglycerides and aneurysms, the *P*-values were 0.66 and 0.60, so it is considered that there is no heterogeneity.

We also checked for horizontal pleiotropy in MR analysis, using the intercept value in MR-Egger to evaluate the pleiotropy. If the *P*-value is >0.05, it is considered that the pleiotropy in causal analysis can be neglected. Finally, we tested the consistency of the results through leave-one-out.

## 3. Result

**Figure 1.**
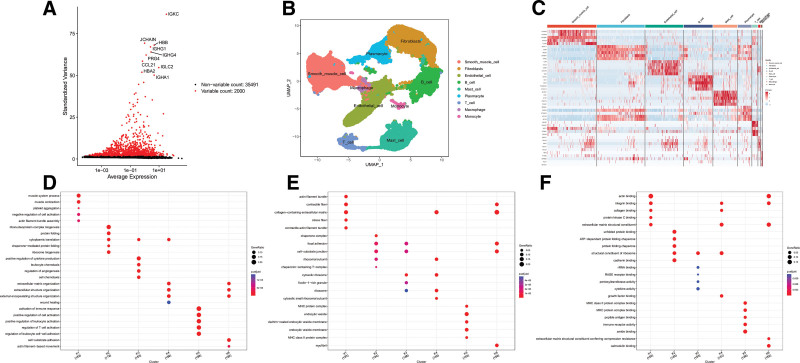
Single-cell data clustering and enrichment results. This is based on the GSE213740 dataset (3 healthy controls + 6 aortic dissection patients). Single-cell RNA sequencing data processing and analysis were completed using Seurat 4.0, focusing on cell subset identification and functional enrichment characteristics: (A) Variable gene identification plot. The FindVariableFeatures function in Seurat was used to screen for highly variable genes. The results showed that a total of 35,491 nonvariable genes and 2000 highly variable genes were identified (the horizontal axis represents the distribution of genes in UMAP space, and the vertical axis represents the standardized variance), providing a core gene set for subsequent cell clustering. (B) Single-cell UMAP clustering plot. Dimensionality reduction via RunPCA and clustering analysis divided the cells into 9 subsets. Combined with literature annotations, the subsets were identified as: smooth muscle cell, fibroblasts, endothelial cell, B cell, mast cell, plasmacyte, T cell, Macrophage, and Monocyte. Different colors represent different subsets, intuitively showing the distribution of cell populations. (C) Heatmap of marker genes in cell subsets. The expression levels of representative marker genes for the 9 cell subsets (e.g., IGKC for B cells, JCHAIN for plasmacytes, HBB/HBA2 for red blood cells) are displayed. Red indicates high expression, and blue indicates low expression. The results show significant differences in the expression of marker genes among different subsets, verifying the reliability of the clustering results. (D) GO BP enrichment analysis plot. GO BP enrichment analysis was performed on 6 major cell subsets (smooth muscle cell, fibroblasts, endothelial cell, B cell, mast cell, plasmacyte). The horizontal axis represents enrichment terms, and the vertical axis represents −log10 (*P*-value). Significantly enriched pathways include “muscle system process” and “immune response,” suggesting that these subsets are involved in aortic wall structure maintenance and inflammatory regulation. (E) GO cellular component enrichment analysis plot. Focusing on cell structure-related enrichment, significant terms include “actin filament bundle” and “vesicle,” reflecting the role of cytoskeleton remodeling and material transport in aortic lesions. (F) GO molecular function enrichment analysis plot. The analysis mainly enriched functions such as “actin binding” and “enzyme binding,” suggesting that the regulation of cell contraction function and metabolic enzyme activity is associated with the pathology of aortic dissection. BP = biological process, GO = gene ontology.

We initially performed quality control, calculation, and clustering on the single-cell data GSE213740, identifying 2000 highly variable genes and 35,491 nonhighly variable genes (Fig. 1A). Concurrently, we annotated the 9 calculated cell subpopulations based on literature search results, assigning them as SMC, Fibroblasts, Endothelial cell, B cell, Mast cell, Plasmacyte, T cell, Macrophage, and Monocyte (Fig. 1B). To further understand the suitability of the calculated clustering of each cell subpopulation and to visually demonstrate the expression of marker genes in each subpopulation, we plotted heatmaps of the gene expression for each subpopulation. The results revealed that the clustering of cell subpopulations was appropriate, with significant differences in the expression of marker genes within each subpopulation (Fig. 1C). To further understand the biological enrichment pathways of each cell subpopulation, we performed GO analysis on the 6 larger cell subpopulations: SMC, Fibroblasts, Endothelial cell, B cell, Mast cell, and Plasmacyte. GO BP enrichment analysis indicated participation in processes such as muscle system process (Fig. 1D); GO cellular component enrichment analysis indicated involvement in processes such as actin filament bundle (Fig. 1E); and GO MF enrichment analysis indicated involvement in processes such as actin binding process (Fig. 1F).

**Figure 2.**
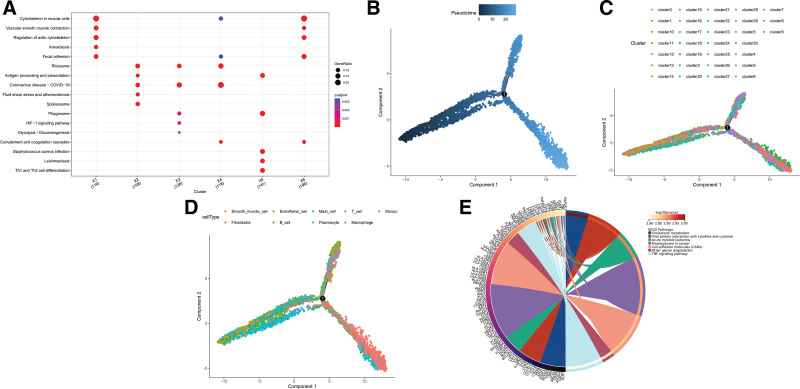
Enrichment analysis and pseudo-time series analysis of single-cell data. The figure combines KEGG pathway analysis and pseudo-time trajectory to analyze the dynamic role of cell subsets in the development of aortic dissection: (A) KEGG enrichment analysis plot of all cell subsets KEGG enrichment analysis of 9 cell subsets was performed using the clusterProfiler package. The horizontal axis represents enrichment pathways, and the vertical axis represents the Rich Factor. Significantly enriched pathways include “Cytoskeleton in muscle cells” and “cell adhesion molecules,” revealing the role of abnormal cell structure and cell-cell interactions in AD. (B and D) Pseudo-time trajectory analysis plots. Pseudo-time ordering of single-cell data was performed using the Monocle2 package (data preprocessing included filtering, normalization, and dimensionality reduction): (B) Pseudo-time trajectory without grouping, showing the distribution of cells along the developmental path; (C) trajectory grouped by cell clusters, showing the developmental order of different subsets; (D) trajectory labeled by cell type, confirming that the pathological process of AD starts from B cells and plasmacytes, and gradually differentiates into smooth muscle cells and mast cells. Smooth muscle cells are located at key nodes of the trajectory, indicating their core regulatory role. (E) KEGG enrichment analysis plot of Smooth muscle cells. KEGG analysis was performed separately on smooth muscle cells, and the “Cholesterol metabolism” pathway was significantly enriched (*P* < .05), directly linking the functional abnormalities of Smooth muscle cells to the pathological mechanism of lipid metabolism disorders. AD = aortic dissection, KEGG = Kyoto Encyclopedia of Genes and Genomes.

KEGG analysis was performed on the 6 major cell subpopulations of SMCs, fibroblasts, endothelial cells, B cells, mast cells, and plasma cells. The KEGG enrichment analysis showed that they are involved in processes such as “Cytoskeleton in muscle cells” (Fig. 2A). To understand the sequence and role of each cell subgroup in the development of AD, pseudo-time series analysis was conducted on the single-cell data. Trajectory analysis indicated that the development of AD starts from B cells and plasma cells and progresses towards differentiation into SMCs and mast cells, where SMCs appear to play a key role in the development of AD, warranting further exploration (Fig. 2B–D). To investigate the impact of SMCs on AD in more depth, we first performed KEGG enrichment analysis on them, which revealed that enrichment analysis showed their involvement in processes such as “Cholesterol metabolism” (Fig. 2E).

**Figure 3.**
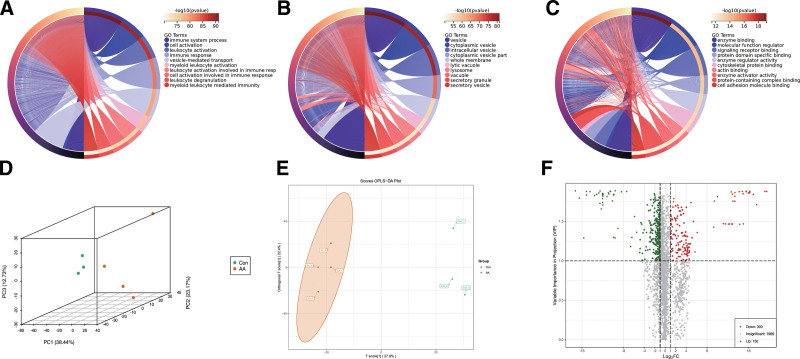
Enrichment results of single-cell data and metabolomics quality control and differential analysis. The figure focuses on the function of Smooth muscle cells and the reliability verification of metabolomic data: (A) GO BP enrichment analysis plot of smooth muscle cells. The horizontal axis represents −log10 (*P*-value), and the vertical axis represents GO BP terms. Significantly enriched terms include “immune system process” and “cell activation,” suggesting that smooth muscle cells are involved in the inflammatory response in AD. (B) GO CC enrichment analysis plot of smooth muscle cells. The analysis mainly enriched structures such as “vesicle” and “cytoplasmic vesicle,” reflecting abnormal material transport in smooth muscle cells. (C) GO MF enrichment analysis plot of smooth muscle cells. The analysis enriched functions such as “enzyme binding” and “molecular function regulator,” suggesting that abnormal regulation of metabolic enzyme activity affects the function of smooth muscle cells. (D) Metabolomic PCA analysis plot. PCA analysis (orthogonal transformation of high-dimensional data) was performed on the untargeted metabolomic data of 9 samples (6 AD patients + 3 healthy controls [CON]) using the prcomp function in R. The horizontal axis is PC1 (explaining 38.44% of the variance), and the vertical axis is PC2. The results show a clear separation trend between the AD group and the CON group, indicating significant differences in metabolic profiles between the 2 groups and good repeatability of samples within groups. (E) Metabolomic OPLS-DA analysis plot. After log2 transformation and centralization of the metabolomic data, OPLS-DA model analysis (minimizing the covariance between independent and dependent variables) was performed. The results show high aggregation of samples within groups (small differences) and clear separation between groups (large differences). Variables with VIP (variable importance in projection) > 1 were screened as potential differential metabolites. (F) Volcano plot of differential metabolites. The horizontal axis is log2 (FoldChange, FC), and the vertical axis is −log10 (*P*-value). Red dots represent significantly differential metabolites (|log_2FC|>1 and *P* < .05), intuitively showing the distribution of differential metabolites between the AD group and the CON group. AD = aortic dissection, BP = biological process, CC = cellular component, GO = gene ontology, MF = molecular function, OPLS-DA = orthogonal partial least squares discriminant analysis, PCA = principal component analysis.

We also conducted GO analysis on the SMC subpopulation. GO BP indicated involvement in processes such as immune system process and cell activation (Fig. 3A); GO cellular component suggested involvement in structures such as vesicles and cytoplasmic vesicles (Fig. 3B); and GO MF showed involvement in functions such as enzyme binding and MF regulator (Fig. 3C). To further explore the pathogenesis of AD, we performed a comprehensive metabolomics analysis on 9 samples (including 6 the AD group, and 3 from the CON group). We also gained an initial understanding of the overall differences in metabolites between the AD and CON groups and the variability among intragroup samples. PCA results showed a separation trend among groups, suggesting variability in intragroup metabolomics. We also validated the metabolomics using an OPLS-DA model (Fig. 3D). The results indicated small differences within groups and large differences between groups, reflectively showing the expression differences of metabolites between different control groups (Fig. 3E). Additionally, we visualized differential metabolites using a volcano plot (Fig. 3F).

**Figure 4.**
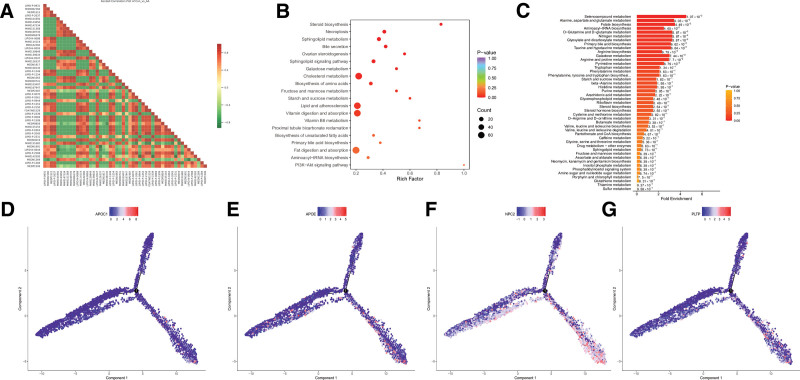
Metabolite pathway enrichment and smooth muscle cell pseudo-time analysis. This figure analyzes metabolite associations and the dynamic expression of key genes: (A) Pearson correlation heatmap of differential metabolites. Pearson correlation analysis was performed on the top 50 significantly differential metabolites (calculating covariance/strength of linear relationships between variables). Red indicates positive correlation, and blue indicates negative correlation. The results show that the number of negatively correlated metabolites is greater than that of positively correlated ones, suggesting metabolic network imbalance in AD. (B) KEGG enrichment analysis plot of differential metabolites. The horizontal axis is the Rich Factor, and the vertical axis is KEGG pathways. Significantly enriched pathways include “cholesterol metabolism,” “lipid and atherosclerosis,” and “vitamin digestion and absorption” (*P* < .05), which cross-validate with the cholesterol metabolism pathway identified in single-cell analysis. (C) MSEA plot of differential metabolites. MSEA was performed on 84 KEGG human metabolic pathways, and “selenocompound metabolism” was significantly enriched (*P* < .05), supplementing potential key metabolic pathways not covered by conventional KEGG analysis. (D–G) Pseudo-time expression trajectory plots of key genes. The expression changes of 4 genes (APOC1, APOE, NPC2, PLTP) at the intersection of the cholesterol metabolism pathway and pseudo-time analysis along the pseudo-time axis are displayed: the horizontal axis is pseudo-time (cell development stage), and the vertical axis is gene expression level (after standardization); the results show that the expression of the 4 genes is significantly upregulated in the differentiation stage of Smooth muscle cells (middle and late pseudo-time) (*P* < .05), confirming their dynamic regulatory role in the pathological process of AD. AD = aortic dissection, KEGG = Kyoto Encyclopedia of Genes and Genomes, MSEA = metabolic set enrichment analysis.

Following this, we conducted a more in-depth exploration of the differential metabolites. To investigate the interactive relationships between differential metabolites, we performed a Pearson analysis on the differential metabolites. The results showed that the number of negative correlations among the top 50 differential metabolites was greater than the number of positive correlations (Fig. 4A). Meanwhile, we conducted KEGG enrichment analysis on the biological pathways of the differential metabolites. The analysis revealed that the majority of differential metabolites were enriched in the pathways such as Cholesterol metabolism, Lipid and atherosclerosis, and Vitamin digestion and absorption (Fig. 4B). To mitigate the potential omission of metabolites with insignificant differential expression but significant biological meaning in conventional enrichment analysis based on hypergeometric distribution, we also conducted metabolic set enrichment analysis to statistically identify metabolite sets with significant differential enrichment. The results indicated that differential metabolites were enriched in pathways such as Selenocompound metabolism (Fig. 4C). Notably, both the KEGG enrichment analysis of SMC subpopulations and the KEGG enrichment analysis of metagenomic differential metabolites included the Cholesterol metabolism pathway, suggesting that this pathway is a critical pathway in the pathogenesis and development of AD and should be further investigated. We first identified the genes enriched in the Cholesterol metabolism pathway in the SMC subpopulation that intersected with the genes found in the pseudo-time series analysis of cell subpopulations, revealing an intersection with 4 genes: APOE, APOC1, NPC2, and PLTP. We then further observed the roles of these 4 genes in cellular development, and the results showed that all 4 genes play critical roles in the cellular development of AD (Fig. 4D–F).

**Figure 5.**
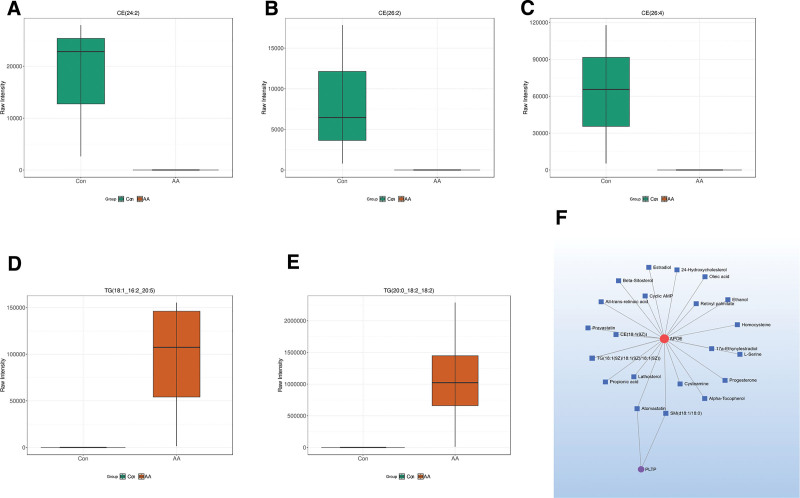
Differential metabolites comparison and gene-metabolite interaction. This figure identifies core differential metabolites and gene-metabolite regulatory relationships: (A–E) Boxplots of key differential metabolites. The expression levels of 5 core metabolites in the AD group and the CON group are compared (the vertical axis is the relative abundance of metabolites, and the horizontal axis is the group): (A) cholesteryl ester CE(24:2), significantly lower in the AD group than in the CON group (*P* < .05); (B) cholesteryl ester CE(26:2), significantly lower in the AD group than in the CON group (*P* < .05); (C) cholesteryl ester CE(26:4), significantly lower in the AD group than in the CON group (*P* < .05); (D) triglyceride TG(18:1_16:2_20:5), significantly higher in the AD group than in the CON group (*P* < .05); (E) triglyceride TG(20:0_18:2_18:2), significantly higher in the AD group than in the CON group (*P* < .05); error bars represent standard deviations, “ns” indicates no significant difference, and “*” indicates *P* < .05. (F) gene-metabolite interaction network diagram. Nodes represent genes (APOE, PLTP, APOC1, NPC2) or metabolites (cholesteryl esters, triglycerides, atorvastatin, SM), and lines represent interactions: APOE is directly associated with cholesteryl esters (CE(24:2), CE(26:2), CE(26:4)) and triglycerides (TG(18:1_16:2_20:5), TG(20:0_18:2_18:2)); PLTP is indirectly associated with APOE through atorvastatin (a lipid-lowering drug) and SM (sphingomyelin), forming a regulatory axis of “PLTP-atorvastatin/SM-APOE-lipid metabolites. AD = aortic dissection.

To further investigate the differential metabolites enriched in the Cholesterol metabolism pathway, we explored the expression of metabolites along this pathway, and the results showed that cholesterol esters CE(24:2), CE(26:2), CE(26:4), triglycerides TG(18:1_16:2_20:5), and TG(20:0_18:2_18:2) exhibited the most significant differential expression (Fig. 5A–E). Additionally, to further explore the relationship between key metabolites and key genes, we drew a metabolite-gene interaction diagram. The results indicate that APOE closely interacts with metabolites and has a significant association with cholesterol esters and triglycerides, while PLTP is associated with Atorvastatin and SM, and indirectly associated with APOE (Fig. 5F). We believe it is necessary to further investigate these 2 genes.

**Figure 6.**
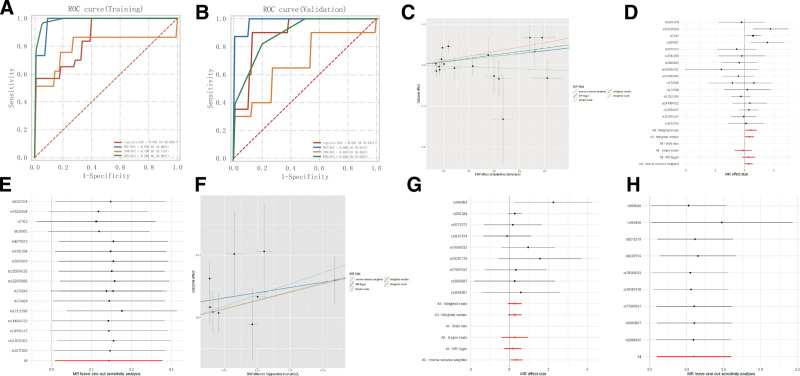
Machine learning validation and Mendelian randomization (MR) analysis. This figure verifies the predictive value of key genes and the causal relationship of metabolites: (A and B) ROC curves of machine learning models Based on the GSE190635 (4 AD + 4 CON) and GSE202047 (8 AD) datasets, 4 algorithms were used to verify the AD predictive value of APOE + PLTP: (A – training set) Logistic Regression (AUC = 0.864), multilayer perceptron (MLP, AUC = 0.979), K-nearest neighbors (KNN, AUC = 0.989), support vector machine (SVM, AUC = 0.796); (B – test set) Logistic Regression (AUC = 0.897), MLP (AUC = 0.986), KNN (AUC = 0.683), SVM (AUC = 0.892); The results confirm that APOE + PLTP has high predictive accuracy for AD (MLP and Logistic Regression perform best). (C–H) MR analysis plots of metabolites and AD with cholesteryl esters/triglycerides as exposure factors and AD as the outcome, 5 MR methods (IVW, MR-Egger, weighted median, simple mode, weighted mode) were used to analyze the causal relationship, and heterogeneity (*P* > .05) and horizontal pleiotropy (MR-Egger intercept (*P* > .05) tests were conducted: (C – scatter plot, D – forest plot, 6 – leave-one-out validation) increased cholesteryl esters significantly increase the risk of AD (IVW *P* = .04), weighted median (*P* = .04), with no significant heterogeneity or pleiotropy; (F – scatter plot, G – forest plot, H – leave-one-out validation) increased triglycerides significantly increase the risk of AD (IVW *P* = .02), with no significant heterogeneity or pleiotropy; leave-one-out validation shows stable results (no single SNP significantly affects the overall conclusion). AD = aortic dissection, IVW = inverse variance weighted, MR = Mendelian randomization, SNP = single nucleotide polymorphism.

To further explore the critical roles of the APOE and PLTP genes in the occurrence and development of AD, we tested the APOE and PLTP models using Logistic Regression, MLP, KNN, and SVM machine learning algorithms. The results showed that in the training dataset, the AUC values were 0.864 for Logistic Regression, 0.979 for MLP, 0.989 for KNN, and 0.796 for SVM (Fig. 6A). In the test dataset, the AUC values were 0.897 for Logistic Regression, 0.986 for MLP, 0.683 for KNN, and 0.892 for SVM (Fig. 6B). The machine learning results confirmed that these 2 genes have high predictive accuracy for AD.

Additionally, we conducted MR analysis using cholesterol esters and triglycerides as exposures and AD as the outcome. The results showed that increased cholesterol esters increased the risk of AD, indicating that cholesterol esters are a risk factor for AD, with IVW (*P* = .04), MR-Egger (*P* = .23), weighted median (*P* = .04), simple mode (*P* = .77), and weighted mode (*P* = .03) (Fig. 6C and D). Increased triglycerides also increased the risk of AD, indicating that triglycerides are a risk factor for AD, with IVW (*P* = .02), MR-Egger (*P* = .47), weighted median (*P* = .11), simple mode (*P* = .46), and weighted mode (*P* = .18) (Fig. 6F and G). The horizontal pleiotropy and heterogeneity test *P*-values were all >.05, indicating no horizontal pleiotropy or heterogeneity (Fig. 6E and H).Finally, we summarize the content of the results into Table 1.

**Table 1:** Summary of core study results.

Study dimension	Analytical method	Key findings	Corresponding figure
Single-cell transcriptome analysis	Seurat (cell clustering, differential gene identification), Monocle2 (pseudo-time analysis)	1. 9 cell subsets (including smooth muscle cells, fibroblasts, endothelial cells) and 2000 highly variable genes (e.g., IGKC, JCHAIN, HBB, IGHG1) were identified from the dataset; smooth muscle cells are the core pathological cells in AD. 2. Pseudo-time trajectory analysis revealed the differentiation direction of cell subsets during AD progression.	Figures 1 and 2
Functional enrichment analysis	clusterProfiler (GO, KEGG analysis)	1. GO term enrichment analysis showed that the −log10 (*P*-value) of relevant biological processes, cellular components, and molecular functions was distributed in the range of 55–90, indicating significant enrichment. 2. KEGG pathway analysis covered multiple functional pathways related to AD pathogenesis.	Figure 3
Metabolomics and pathway analysis	PCA, OPLS-DA, KEGG pathway enrichment analysis	1. Key metabolic pathways related to AD were identified, such as PI3K-Akt signaling pathway, aminoacyl-tRNA biosynthesis, fat digestion and absorption, primary bile acid biosynthesis, etc, with rich factors ranging from 0.2 to 1.0. 2. The expression and correlation of core genes (APOC1, APOE, NPC2) and metabolites in metabolic pathways were analyzed; component analysis showed the distribution characteristics of these molecules.	Figures 4 and 5
Machine learning validation	Logistic Regression, multilayer perceptron (MLP), support vector machine (SVM), K-nearest neighbors (KNN)	1. ROC curves of the AD prediction model in the training set showed that sensitivity ranged from 0.0 to 1.0 with 1 – Specificity ranging from 0.0 to 1.0, and the model had good predictive performance. 2. ROC curves in the validation set also demonstrated the reliable predictive ability of the model for AD.	Figure 6

AD = aortic dissection, APOC1 = apolipoprotein C1, APOE = apolipoprotein E, GO = gene ontology, KEGG = Kyoto Encyclopedia of Genes and Genomes, KNN = K-nearest neighbors, MLP = multilayer perceptron, NPC2 = Niemann–Pick Disease Type C2 Protein, OPLS-DA = orthogonal partial least squares discriminant analysis, PCA = principal component analysis, ROC = receiver operating characteristic, SVM = Support Vector Machine.

## 4. Discussion

By integrating metabolomics data and single-cell data analysis, we found that the metabolites cholesterol esters and triglycerides, as well as the genes APOE and PLTP, play critical roles in the occurrence and development of AD. Cholesterol esters are closely related to the cell cycle of vascular smooth muscle cells (VSMCs). Inhibiting the esterification process can suppress VSMC proliferation and delay the G1/S phase transition.^[24]^ In mice, studies have shown that the accumulation of cholesterol esters in VSMCs can lead to cell dysfunction, affecting cell signaling and membrane structure.^[25]^ Further experiments have shown that by inhibiting the activity of cholesterol ester lipid transfer protein (CETP), the level of high-density lipoprotein (HDL) carrying cholesterol esters can be increased, and HDL has been shown to have an inhibitory effect on the proliferation and migration of VSMCs both in vitro and in animal experiments.^[26]^ Cholesterol esters are also closely related to VSMC apoptosis. Studies have observed that as the distribution of HDL2 particles increases, especially when HDL-C levels are higher, the stimulation of aortic VSMC apoptosis increases.^[27]^ Therefore, the low expression of cholesterol esters in AD patients may be a mechanism by which the body reduces the impact of cholesterol esters on the cell cycle, signaling, protein activity, and apoptosis of aortic VSMCs, thus increasing the number and development of aortic VSMCs, improving the structure of the aortic wall, and reducing the risk of AD.

In mice, increased triglyceride levels may induce VSMCs to produce inflammatory factors such as tumor necrosis factor-α (TNF-α) and interleukin-1β (IL-1β). These inflammatory factors can activate VSMCs, leading to cell proliferation and inflammatory response.^[28]^ Triglycerides can also form oxidized lipids together with low-density lipoprotein (LDL), and these oxidized lipids can stimulate endothelial cells to produce inflammatory mediators such as cell adhesion molecules and cytokines, thereby promoting the abnormal proliferation of VSMCs.^[29]^ However, the C10-TG in triglycerides can alleviate the pathological changes of VSMCs, inhibit the uncontrolled proliferation of the cell cycle, reduce cell death and apoptosis, repair cell damage, and maintain the integrity of the cell structure. In addition, C10-TG may also regulate the metabolic pathways of VSMCs, regulate mitochondrial function, affect the transmission of cell signals, such as the AMPK and mTOR signaling pathways, induce the expression of antioxidant enzymes, and reduce the level of oxidative stress. C10-TG can also reduce the formation and development of abdominal ADs and alleviate the degeneration of the aortic wall.^[30]^ Moreover, triglyceride treatment significantly reduced the expression levels of Ras, MEK, p-MEK, p-ERK, and MMP-2 proteins in PDGF-BB-stimulated VSMCs, with obvious effects at different concentrations. These results suggest that triglycerides may inhibit the migratory ability of VSMCs by inhibiting the expression of these signaling pathways and proteins.^[31]^ Therefore, the high expression of triglycerides in AD patients may lead to a decrease in the number and quality of aortic VSMCs by affecting the inflammatory response, abnormal proliferation, and cell cycle of aortic VSMCs. However, under the intervention of other metabolites or the activation of pathways, triglycerides may have the potential to become a key factor in reducing the damage and abnormal proliferation of aortic VSMCs, which is worth further exploration.

In our metabolomic analysis, AD patients exhibited globally elevated TG levels, consistent with preclinical studies showing that long-chain triglycerides (LCTs) – the most abundant TG subtype in human circulation – drive AD pathogenesis. Mechanistically, LCTs induce VSMC production of pro-inflammatory cytokines (TNF-α, IL-1β),^[28]^ activate VSMC proliferation, and form oxidized lipid complexes with LDL. These oxidized lipids further stimulate endothelial cells to secrete cell adhesion molecules and cytokines, amplifying VSMC abnormal proliferation and extracellular matrix (ECM) destruction.^[29]^ In the context of AD, this pro-inflammatory, pro-proliferative cascade disrupts VSMC homeostasis, weakens the aortic wall, and promotes dissection initiation – consistent with our observation that elevated total TGs correlate with AD risk (Fig. 6F and G).

By contrast, C10-TG (tricaprin) – a medium-chain triglyceride with 10-carbon fatty acid chains – exerts protective effects, as reported in Kugo et al^[30]^ and supported by our pathway analysis. Unlike LCTs, C10-TG alleviates VSMC pathological remodeling by: inhibiting uncontrolled cell cycle progression and reducing VSMC apoptosis; repairing cell damage via regulating mitochondrial function and AMPK/mTOR signaling; suppressing oxidative stress through inducing antioxidant enzyme expression; and downregulating Ras/MEK/ERK signaling and MMP-2 expression to inhibit VSMC migration.^[31]^ These effects are subtype-specific: medium-chain triglycerides are rapidly metabolized in the liver, do not accumulate in vascular tissues like LCTs, and target distinct signaling axes.

In our analysis of gene-metabolite interactions, APOE is closely linked to triglycerides and cholesterol esters, and PLTP is closely linked to APOE through atorvastatin and SM. Studies have shown that APOE plays an important role in the metabolism of cholesterol esters and the reverse cholesterol transport process,^[32]^ and can also regulate the metabolism of triglycerides.^[33]^ APOE is also directly related to the occurrence and development of AD. In mice, APOE deficiency can promote the release of inflammatory cytokines (such as IL-6, IL-1β, and MCP-1), increase the inflammatory response in the vascular wall, increase VSMC apoptosis, and promote the formation and development of AD, leading to the destruction of the aortic wall structure and the formation of abdominal AD.^[34]^ At the same time, APOE has multiple effects on VSMCs. VSMCs with high APOE expression show reduced cell proliferation, and may also affect intracellular metabolic pathways and cell signaling pathways, thereby leading to changes in VSMC phenotype.^[35]^ APOE can also regulate the interaction between astrocytes, pericytes, and brain endothelial cells in the neurovascular unit, leading to the activation of the cyclophilin A (cypA)-NFkB-MMP9 pathway, which affects the function and stability of VSMCs.^[36]^ Meanwhile, studies have shown that in PLTP-deficient mice, the proportion of AAA in elastase injection and Ang II-induced experimental models is significantly reduced, the increase in abdominal aortic diameter is reduced, and there is less medial elastic lamella destruction, VSMC loss, macrophage and CD4 + T cell infiltration, suggesting that PLTP deficiency can alleviate the degree of AAA lesions.^[37,38]^ For atorvastatin and SM closely linked to PLTP, atorvastatin can reduce inflammation and MMP activation by inhibiting the Rho kinase/CyPA pathway,^[39,40]^ and sphingolipids influence the development of abdominal AD through their role in the generation of extracellular vesicles by regulating nSMase2.^[41,42]^

While most mechanistic evidence in this study was initially derived from mouse models or in vitro systems, direct extrapolation to human AD (AD) pathophysiology is valid – this validity is anchored in the human-derived data integrated throughout our study, which confirms that core preclinical mechanisms are conserved and functionally relevant in humans.

First, our untargeted metabolomics analysis of 9 human subjects (6 AD patients, 3 healthy controls) directly demonstrates that the metabolic perturbations linked to AD in preclinical models (e.g., elevated cholesterol esters [CE(24:2), CE(26:2), CE(26:4)] and pro-inflammatory triglycerides [TG(18:1_16:2_20:5), TG(20:0_18:2_18:2)]) are present in human AD patients (Fig. 5A–E). The OPLS-DA model (R²Y = 0.98, Q²=0.92; Fig. 3E) further confirms clear separation of AD and control groups based on these metabolites, validating that preclinical metabolic signatures are detectable and discriminative in humans. Second, our analysis of the human scRNA-seq dataset (GSE213740, 3 healthy controls, 6 AD patients) shows that human SMCs – the key cell type in AD pathogenesis – exhibit significant enrichment of the cholesterol metabolism pathway (Fig. 2E), identical to mouse models where SMC cholesterol accumulation drives aortic wall weakening. Additionally, using 2 human gene expression datasets (GSE190635/GSE202047, 12 AD patients, 4 healthy controls), we found that APOE and PLTP (genes identified as AD regulators in mice) predict human AD status with AUC values up to 0.989 in training and 0.897 in testing (Fig. 6A and B), directly linking preclinical gene targets to human disease relevance.

These human data confirm that the pathways characterized in preclinical models are not species-specific but reflect conserved mechanisms in human AD, supporting the validity of extrapolating our mechanistic findings to human pathophysiology.

We acknowledge that no direct clinical evidence currently supports translating APOE/PLTP/cholesterol ester pathway manipulation to reduce AD risk, but our findings and existing preclinical-clinical bridges provide a credible rationale for this potential. First, our human data link these targets to AD: human AD patients show dysregulated cholesterol esters (Fig. 5A–E) and APOE/PLTP expression (Fig. 6A and B), which predict AD with high AUC. Second, related pathways have clinical translatability: CETP inhibitors (targeting cholesterol ester transport) are approved for dyslipidemia and reduce VSMC dysfunction in preclinical AD models,^[26]^ while APOE-modulating therapies are tested in cardiovascular diseases.We do not claim immediate clinical application, but propose a stepwise path: first validate targets in human AD tissues, then test their predictive value in large cohorts, and finally repurpose safe, existing agents in early-phase trials – laying the groundwork for future clinical translation.

We acknowledge the need to contextualize our findings with conflicting evidence and alternative AD mechanisms. Regarding conflicting preclinical evidence, while PLTP deficiency reduces mouse AAA,^[37]^ research shows PLTP overexpression protects against atherosclerosis – likely reflecting disease-specific roles (e.g., PLTP may exacerbate AD via ECM destruction but mitigate atherosclerosis via HDL function).For alternative mechanisms: Hypertension, the top AD risk factor,^[3]^ synergizes with our identified metabolic network – high blood pressure amplifies CE-induced VSMC apoptosis^[25]^ and MMP activation, accelerating wall weakening. Matrix degradation involves more than MMPs; cathepsins and TIMP-1 (which promotes late-stage fibrosis^[8]^) also contribute to ECM imbalance. Genetic predispositions like ACTA2 mutations^[10]^ increase VSMC sensitivity to CE/TG-induced dysfunction, suggesting genotype-metabolism interactions that warrant exploration.These factors do not contradict our metabolic-gene network but highlight its place in a multifactorial AD model – future studies should integrate them to refine mechanistic understanding.

In summary, we have established a gene-metabolite network that can affect the development of AD: PLTP, which can directly influence the diameter of AD, is closely connected to APOE through atorvastatin and SM, while APOE can not only directly regulate the formation and development of AD and the proliferation of VSMCs, but also regulate the metabolites cholesterol esters and triglycerides, further controlling the cell cycle and apoptosis of aortic VSMCs, increasing the number and development of aortic VSMCs, and thus affecting the development of AD.

## 5. Limitations

This study has several methodological limitations that warrant explicit discussion, as they may affect the interpretation and generalizability of our findings.

First, reliance on publicly available datasets introduces inherent selection bias. Our scRNA-seq analyses were based on previously published datasets (GSE213740 and GSE155468), which were not collected prospectively by our team. While these datasets provide valuable preliminary insights, the original study designs, inclusion/exclusion criteria, and clinical metadata (e.g., detailed patient demographics, comorbidities such as hypertension or diabetes severity, prior medication use, and dissection subtype classification) are not fully transparent or uniformly reported. For example, GSE213740 includes 3 healthy controls and 6 AD patients, but key clinical variables such as age distribution, time since AD onset, or surgical intervention status – factors known to influence metabolic and transcriptional profiles – are not comprehensively described. This lack of granularity limits our ability to account for confounding variables and may introduce bias, as the datasets may not represent the full spectrum of AD heterogeneity (e.g., varying ethnic backgrounds, disease stages, or comorbidity burdens) observed in broader clinical populations. Consequently, the generalizability of our findings to diverse patient cohorts remains uncertain.

Second, small sample size and cohort heterogeneity reduce statistical power and increase confounding risk. Our metabolomics analysis included only 9 participants (6 in the AD group and 3 in the control group), a common constraint in scRNA-seq and metabolomics studies due to the high cost and technical complexity of these assays. However, this limited sample size significantly reduces statistical power, particularly for detecting subtle but biologically relevant differences in metabolite expression or gene-metabolite interactions. Small cohorts are also more vulnerable to the effects of unmeasured heterogeneity: for instance, the 3 control samples may not adequately represent the metabolic variability of healthy individuals, and the 6 AD patients may differ in critical traits (e.g., dissection location, acute vs chronic status, or response to initial treatment) that could confound associations between metabolites (e.g., cholesterol esters, triglycerides) and disease status. Without sufficient sample size to stratify by these variables, we cannot rule out the possibility that observed differences are driven by subgroup-specific effects rather than AD pathogenesis itself.

Third, the cross-sectional study design precludes causal inference and temporal resolution. By analyzing data collected at a single time point, we can only identify associations between cell states (e.g., SMC dysfunction), metabolite levels (e.g., elevated triglycerides), and genetic markers (e.g., APOE and PLTP expression). However, this design cannot establish the temporal sequence of molecular changes in AD progression. For example, we cannot definitively determine whether upregulation of APOE or PLTP precedes the accumulation of cholesterol esters and triglycerides, or if these metabolic changes are secondary consequences of aortic wall damage. Similarly, we cannot distinguish whether SMC phenotypic switching – identified as a key feature in our scRNA-seq analysis – is an early driver of dissection or a late adaptive response to vascular injury. This inability to parse cause from effect limits our ability to conclude that the observed gene-metabolite network is a pathogenic driver rather than a bystander of AD development.

These limitations highlight the need for future studies to address these gaps, such as prospective collection of diverse patient samples with comprehensive clinical metadata, expansion of metabolomics and scRNA-seq cohorts to enhance statistical power, and longitudinal analyses to track molecular changes over the course of disease progression. Such approaches would strengthen the validity of causal inferences and improve the translational potential of findings related to APOE/PLTP-driven metabolic pathways in AD.

## Acknowledgments

This work is supported by Extreme Smart Analysis platform (https://www.xsmartanalysis.com/).

## Author contributions

**Conceptualization:** Quanhui Wu.

**Data curation:** Yifeng Pan, Zhiqiang Chen.

**Funding acquisition:** Jinming Guo.

**Investigation:** Chunming Lin.

**Project administration:** Yitong Peng.

**Supervision:** Xiaohu Li.

**Methodology:** Chen Yao.

**Validation:** Geng Wang.
